# Efficacy and Safety of Efinaconazole 10% Topical Solution for Treatment of Onychomycosis in Older Adults: A Post Hoc Analysis of Two Phase 3 Randomised Trials

**DOI:** 10.1111/myc.70069

**Published:** 2025-05-21

**Authors:** Shari R. Lipner, Aditya K. Gupta, Warren S. Joseph, Boni Elewski, Eric Guenin, Tracey C. Vlahovic

**Affiliations:** ^1^ Weill Cornell Medicine New York New York USA; ^2^ Mediprobe Research Inc. London Ontario Canada; ^3^ Division of Dermatology, Temerty Faculty of Medicine, Department of Medicine University of Toronto Toronto Ontario Canada; ^4^ Arizona College of Podiatric Medicine Midwestern University Glendale Arizona USA; ^5^ University of Alabama at Birmingham School of Medicine Birmingham Alabama USA; ^6^ Ortho Dermatologics, a division of Bausch Health US, LLC Bridgewater New Jersey USA; ^7^ Samuel Merritt University College of Podiatric Medicine Oakland California USA

**Keywords:** clinical trial, efinaconazole, elderly, onychomycosis, toenail, topical

## Abstract

**Background:**

Onychomycosis is common in older adults and can be difficult to treat owing to slower nail growth, increased nail thickness, comorbidities, and concomitant medications. Oral treatments can be complicated by contraindications, drug–drug interactions, and adverse effects. Topical treatments such as efinaconazole 10% solution may be beneficial for treating older adults.

**Objectives:**

To evaluate the efficacy/safety of efinaconazole 10% solution in adults aged ≥ 65 years with toenail onychomycosis.

**Patients/Methods:**

In two multicenter, double‐blind, phase 3 studies (NCT01008033; NCT01007708), patients with mild to moderate toenail onychomycosis were randomised (3:1) to once‐daily efinaconazole or vehicle for 48 weeks, with a 4‐week follow‐up. Pooled data for participants aged ≥ 65 years were analysed post hoc (*n* = 162 efinaconazole, *n* = 56 vehicle). The primary endpoint was complete cure (0% involvement of target toenail plus mycologic cure [negative KOH and fungal culture]) at week 52. Treatment‐emergent adverse events (TEAEs) were assessed throughout.

**Results:**

At week 52, a significantly greater proportion of older adults (aged 65–71 years) achieved complete cure with efinaconazole than vehicle (13.6% vs. 3.6%; *p* < 0.05). Complete/almost complete cure rate was also significantly greater (≤ 5% involvement and mycologic cure; 19.1% vs. 5.4%; *p* = 0.01), and over half (59.2%) of participants achieved mycologic cure with efinaconazole versus 12.5% with vehicle (*p* < 0.001). Treatment‐related TEAE rates with efinaconazole were low (6.0%) and similar to the overall study population.

**Conclusions:**

Efinaconazole 10% solution showed similar efficacy/safety in participants aged ≥ 65 years to the overall phase 3 population, despite potential age‐related nail changes. These results demonstrate the benefits of efinaconazole in older patients with onychomycosis.

## 1 Introduction

Onychomycosis is a common, chronic fungal infection of the nail plate or bed that is caused by dermatophytes, nondermatophyte moulds (NDM), or yeasts [1]. Although it is a benign condition, onychomycosis can cause discomfort and pain and decreased quality of life; if untreated, it can lead to secondary infections and even amputations, particularly in diabetics [2]. Treatment of onychomycosis can be challenging because of reduced drug penetration owing to the thick nail plate, potential drug–drug interactions and side effects, and lack of patient compliance [1, 2, 3, 4]. Onychomycosis is more common in older adults [5], who may face additional therapeutic challenges due to slower nail growth, increased nail thickness, longer disease duration, comorbidities such as diabetes or peripheral vascular disease, and concomitant medication use [3, 6, 7, 8]. Additionally, older adults may have reduced visual acuity and dexterity that could reduce compliance with topical treatments [3, 5].

Oral terbinafine is often prescribed as a first‐line treatment for onychomycosis due to its good efficacy and low cost [9, 10, 11]. However, as rates of antifungal resistance rise globally, terbinafine may not be effective for all patients [12, 13]. Other oral treatments approved in the US include itraconazole and griseofulvin, with fluconazole being prescribed off‐label [1]. Griseofulvin is rarely prescribed due to its inferior efficacy and greater recurrence rates compared with other oral treatments, and itraconazole has black box warnings for congestive heart failure, cardiac effects, and multiple drug–drug interactions [14, 15]. Furthermore, the use of oral medications in older adults can be complicated by contraindications, interactions with concomitant medications, and adverse effects, such as cardiac issues, hepatotoxicity, or liver enzyme abnormalities [15, 16, 17, 18].

A 2021 therapeutic recommendations publication suggests that topical efinaconazole 10% solution may be beneficial as a first‐line treatment for most patients with onychomycosis, either as monotherapy or in combination with an oral antifungal [1]. In particular, for patients aged 65 years and older, combination therapy with a topical such as efinaconazole and an oral agent such as terbinafine or fluconazole is recommended. When interactions with concomitant medications are a concern, efinaconazole alone may be preferable.

In two phase 3 clinical trials, topical efinaconazole 10% demonstrated good efficacy and safety in patients with mild to moderate toenail onychomycosis [19]. Here, we report post hoc analyses evaluating the efficacy and safety of once‐daily topical efinaconazole 10% solution in older adult study participants (aged ≥ 65 years).

## 2 Methods

### 2.1 Study Design and Participants

Data were pooled from two multicenter, randomised, double‐blind, vehicle‐controlled, parallel‐group, phase 3 studies (NCT01008033 and NCT01007708), the details of which have been published previously [19]. Briefly, eligible participants aged 18–70 years with mild to moderate toenail distal lateral subungual onychomycosis (20%–50% clinical involvement of the target toenail and no matrix involvement or dermatophytomas) affecting at least one great toenail were randomised (3:1) to topical efinaconazole 10% solution (Jublia; Ortho Dermatologics, Bridgewater, NJ) or vehicle solution self‐applied once daily for 48 weeks. After a 4‐week treatment‐free period, a follow‐up visit was conducted at week 52. To evaluate the efficacy and safety of efinaconazole in older adults, a pooled post hoc analysis was conducted in participants aged ≥ 65 years.

### 2.2 Ethics

The studies were conducted in accordance with the principles of Good Clinical Practice and the Declaration of Helsinki, and study protocols were approved by the ethics committee or institutional review board at all sites of investigation. All participants provided written informed consent prior to participation in the study.

### 2.3 Assessments

Efficacy was determined by a combination of visual assessment of the percentage of affected toenail area (i.e., clinical involvement), potassium hydroxide (KOH) examination, and fungal culture of toenail samples taken at baseline and at weeks 12, 24, 36, 48, and 52. The primary efficacy endpoint was the rate of complete cure (no clinical involvement, negative KOH examination, and negative fungal culture of the target toenail) at week 52. Secondary efficacy endpoints included rates of mycologic cure (a negative KOH examination and a negative fungal culture of the target toenail), complete or almost complete cure (≤ 5% clinical involvement and mycologic cure), unaffected new toenail growth (change from baseline in the healthy/unaffected target toenail measurement), and clinical efficacy (< 10% clinical involvement of the affected target nail) at week 52.

Participants also completed the OnyCOE‐t^tm^ quality of life (QoL) questionnaire [20] at baseline, week 24, and week 52. The questionnaire comprises 33 questions across seven scales that assess toenail symptoms, problems with appearance, problems with physical activities, overall problems, stigma, and treatment satisfaction.

Adverse events (AEs) were monitored throughout the study.

### 2.4 Statistical Analysis

The intent‐to‐treat (ITT) population included all randomised participants who were provided the study drug. The safety population included all randomised participants who used the study drug at least once and had at least one post‐baseline assessment. Post hoc analyses assessed only participants aged 65 years and older.

Efficacy endpoints at week 52 were analysed using Cochran–Mantel–Haenszel tests stratified by analysis center at a significance level of 5%. Interpretation of the secondary endpoints of unaffected toenail growth and mycologic cure was performed in a sequential manner to adjust for multiplicity. Unaffected new toenail growth was considered statistically significant (active vs. vehicle) only if the complete or almost complete cure rate was statistically significant. The mycologic cure rate was considered statistically significant (active vs. vehicle) only if both the complete or almost complete cure rate and unaffected new toenail growth were statistically significant. The last observation carried forward method was used to impute missing efficacy data. All statistical analyses were performed using SAS. Statistical significance was only determined for week 52. Efficacy outcomes at weeks 12–48 were summarised using descriptive statistics.

Treatment compliance (defined as not missing > 14 cumulative doses in the 28 days leading up to week 48, not missing > 20% of the total number of expected doses during treatment, and/or not missing ≥ 28 consecutive doses during treatment), safety, and QoL assessments were summarised using descriptive statistics. All items in the OnyCOE‐t QoL questionnaire were transformed to a 0 to 100 scale; higher scores indicated improved QoL.

All AEs were recorded and classified using Medical Dictionary for Regulatory Activities (version 12.1) terminology. No imputations were performed for missing safety data.

## 3 Results

### 3.1 Participant Disposition and Demographics

Of the 1655 participants randomised in the two phase 3 studies [19], 218 were aged 65–71 years (*n* = 162 efinaconazole, *n* = 56 vehicle). The majority were male (75.2%), and most were White (72.0%) and not Hispanic/Latino (90.4%; Table 1). The mean area of target toenail involvement was 37.8%, and the mean number of affected nontarget toenails was 2.9. Approximately 89% of participants were treatment compliant.

**TABLE 1:** Demographic and baseline characteristics in patients aged ≥ 65 years (ITT population, pooled).

	Efinaconazole 10% (*n* = 162)	Vehicle (*n* = 56)
Age, mean (range), year	67.5 (65.0–71.0^a^)	67.1 (65.0–70.0)
Male, *n* (%)	123 (75.9)	41 (73.2)
Race, *n* (%)		
Asian	43 (26.5)	11 (19.6)
Black	3 (1.9)	2 (3.6)
White	114 (70.4)	43 (76.8)
Other^b^	2 (1.2)	0
Ethnicity, *n* (%)		
Not Hispanic or Latino	149 (92.0)	48 (85.7)
Affected toenail, mean (range), %	37.8 (20.0–50.0)	37.9 (20.0–50.0)
Affected nontarget toenails, mean (range), *n*	2.8 (0–5.0)	3.3 (0–5.0)

Abbreviation: ITT, intent to treat.

^a^ Participants may have turned 71 years of age by the date of the baseline visit, which occurred 42 days after screening owing to mycological testing. An additional 42‐day period could have occurred if further tests were required.

^b^ American Indian/Alaskan Native, Native Hawaiian/Pacific Islander, and Other.

### 3.2 Efficacy

**FIGURE 1:**
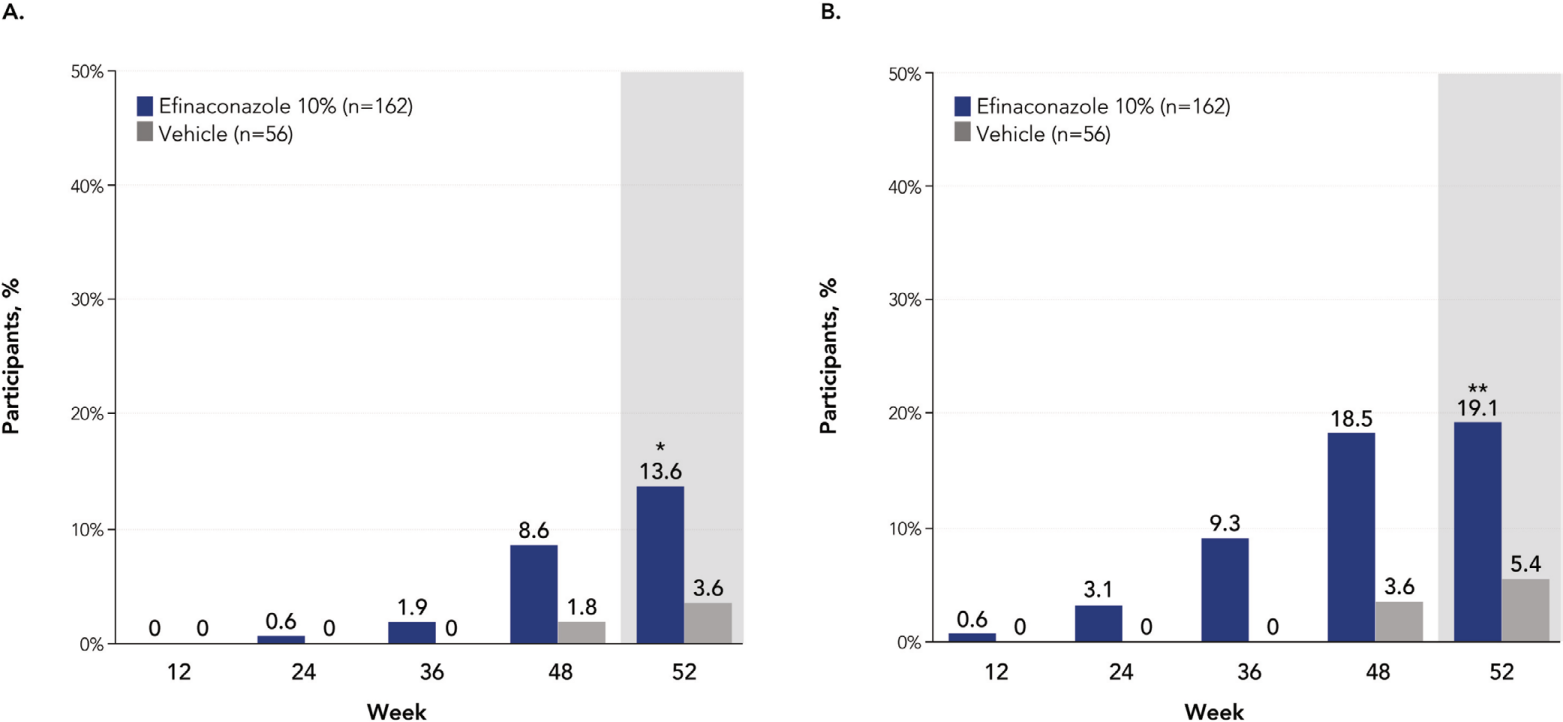
Complete cure (no clinical involvement and mycologic cure [negative KOH examination and negative fungal culture] of the target toenail) (A) and complete or almost complete cure (≤ 5% clinical involvement and mycologic cure [negative KOH examination and negative fungal culture] of the target toenail) (B) by visit in patients aged ≥ 65 years (ITT population, pooled). *p < 0.05; **p = 0.01 vs. vehicle. Statistical significance was only determined for week 52 because the study was not powered for subgroup analyses. Complete cure and complete/almost complete cure rates at week 52 with efinaconazole in the overall phase 3 populations were 17.8% and 15.2% and 26.4% and 23.4%, respectively [19]. ITT, intent to treat; KOH, potassium hydroxide.

**FIGURE 2:**
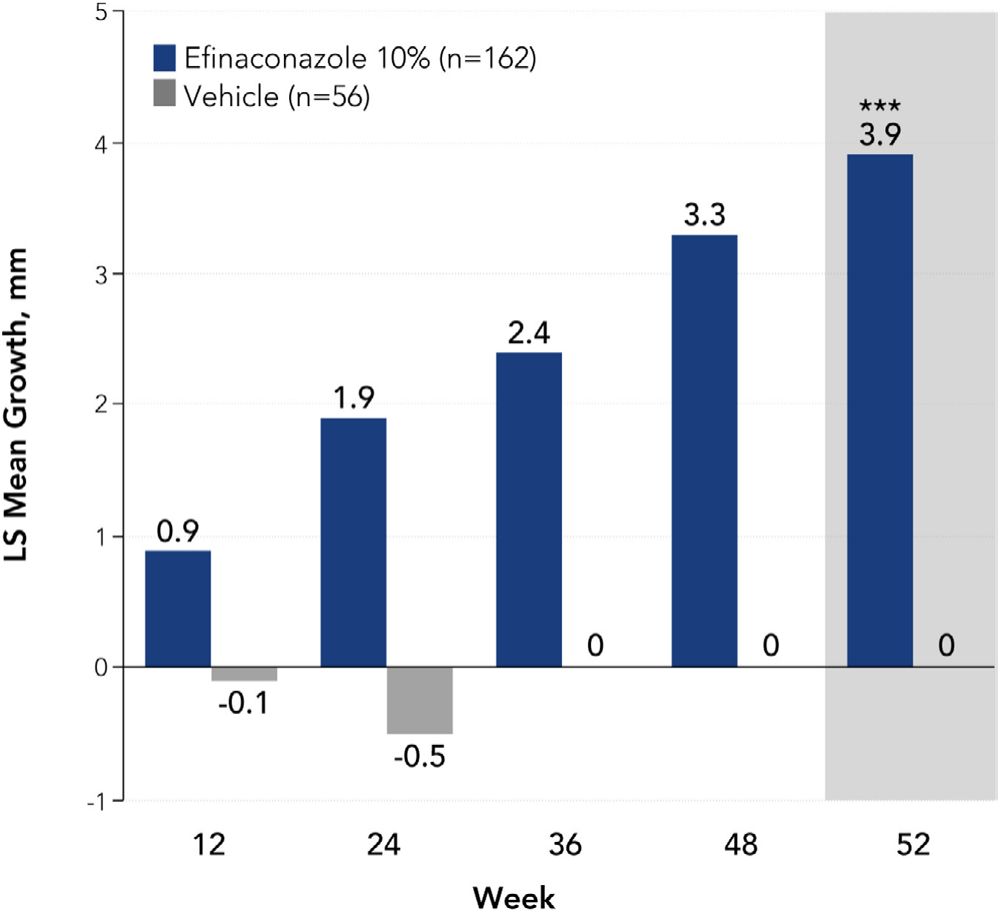
Unaffected new toenail growth (change from baseline in the healthy [unaffected] toenail measurement of the target toenail) by visit in patients aged ≥ 65 years (ITT population, pooled). ***p < 0.001 vs. vehicle. Statistical significance was only determined for week 52 because the study was not powered for subgroup analyses. Mean unaffected new toenail growth at week 52 with efinaconazole in the overall phase 3 populations was 5.0 mm and 3.8 mm [19]. LS, least squares; ITT, intent to treat.

**FIGURE 3:**
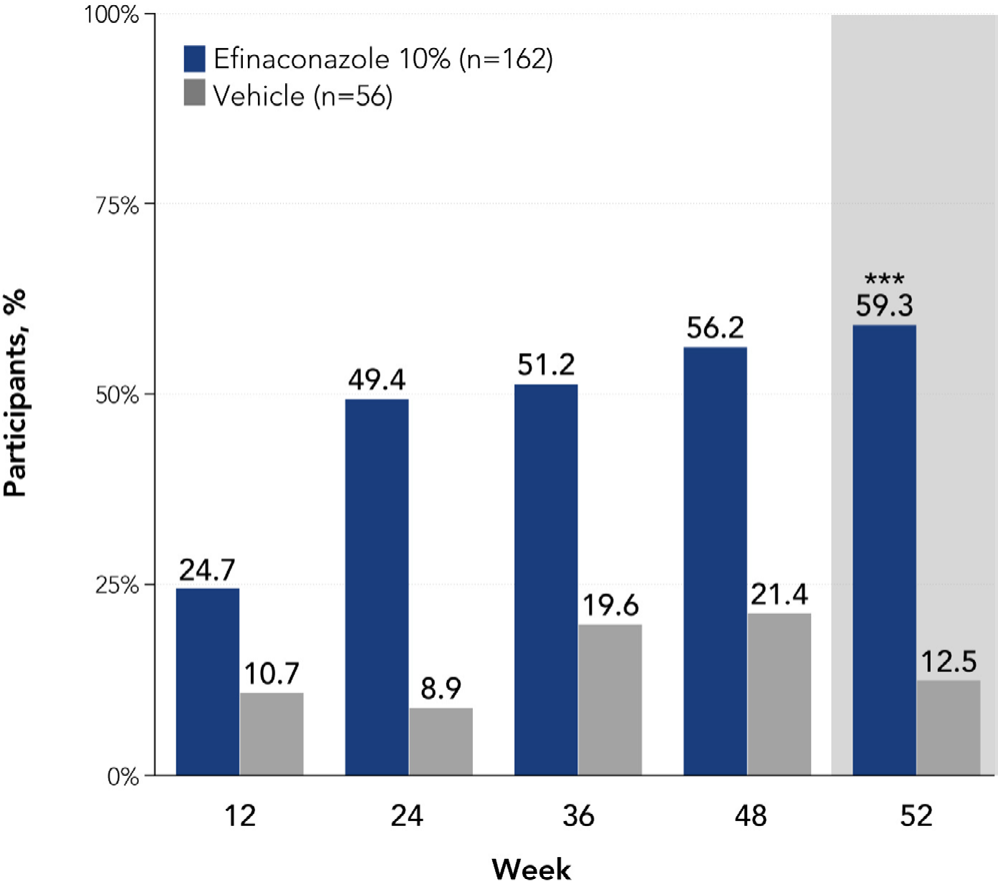
Mycologic cure (negative KOH examination and negative fungal culture of the target toenail) by visit in patients aged ≥ 65 years (ITT population, pooled). ***p < 0.001 vs. vehicle. Statistical significance was only determined for week 52 because the study was not powered for subgroup analyses. Interpretation was performed in a sequential manner to adjust for multiplicity: Mycologic cure rate was considered statistically significant only if complete/almost complete cure rate *and* unaffected new toenail growth were significant. Mycologic cure rates at week 52 with efinaconazole in the overall phase 3 populations were 55.2% and 53.4% [19]. ITT, intent to treat; KOH, potassium hydroxide.

**FIGURE 4:**
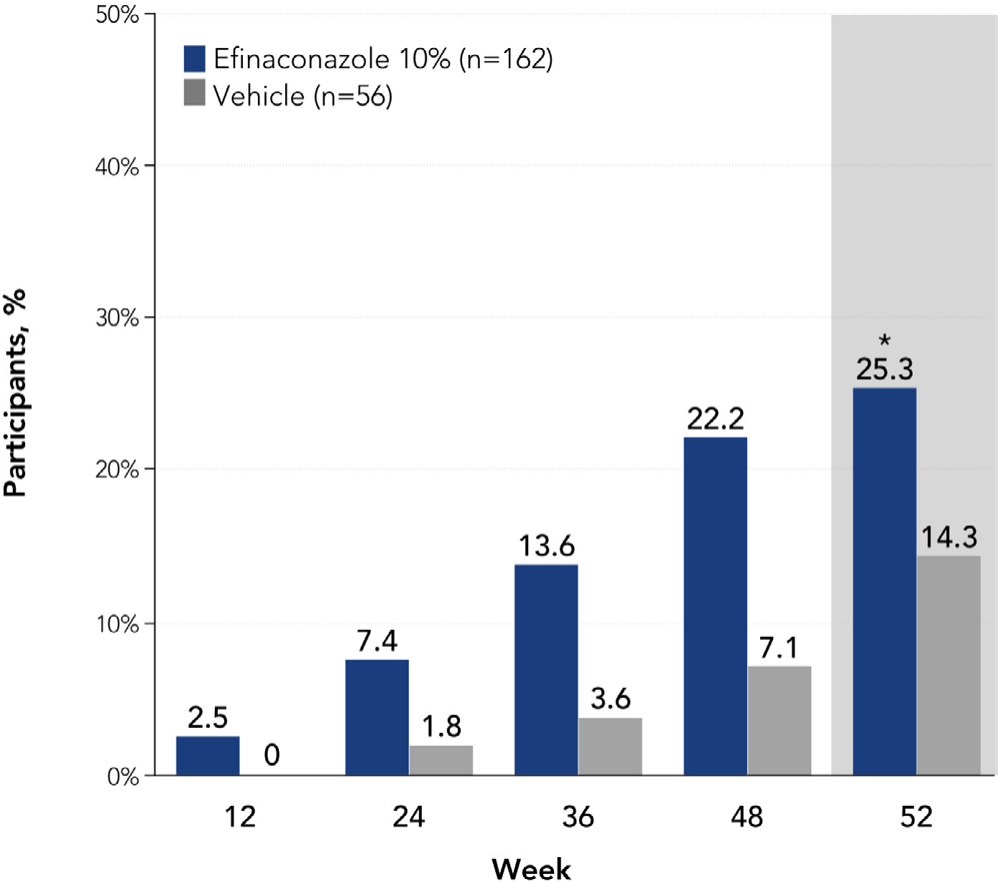
Clinical efficacy (< 10% involvement of the target toenail) by visit in patients aged ≥ 65 years (ITT population, pooled). *p < 0.05 vs. vehicle. Statistical significance was only determined for week 52 because the study was not powered for subgroup analyses. Clinical efficacy rates at week 52 with efinaconazole in the overall phase 3 populations were 35.7% and 31.0% [19]. ITT, intent to treat.

**FIGURE 5:**
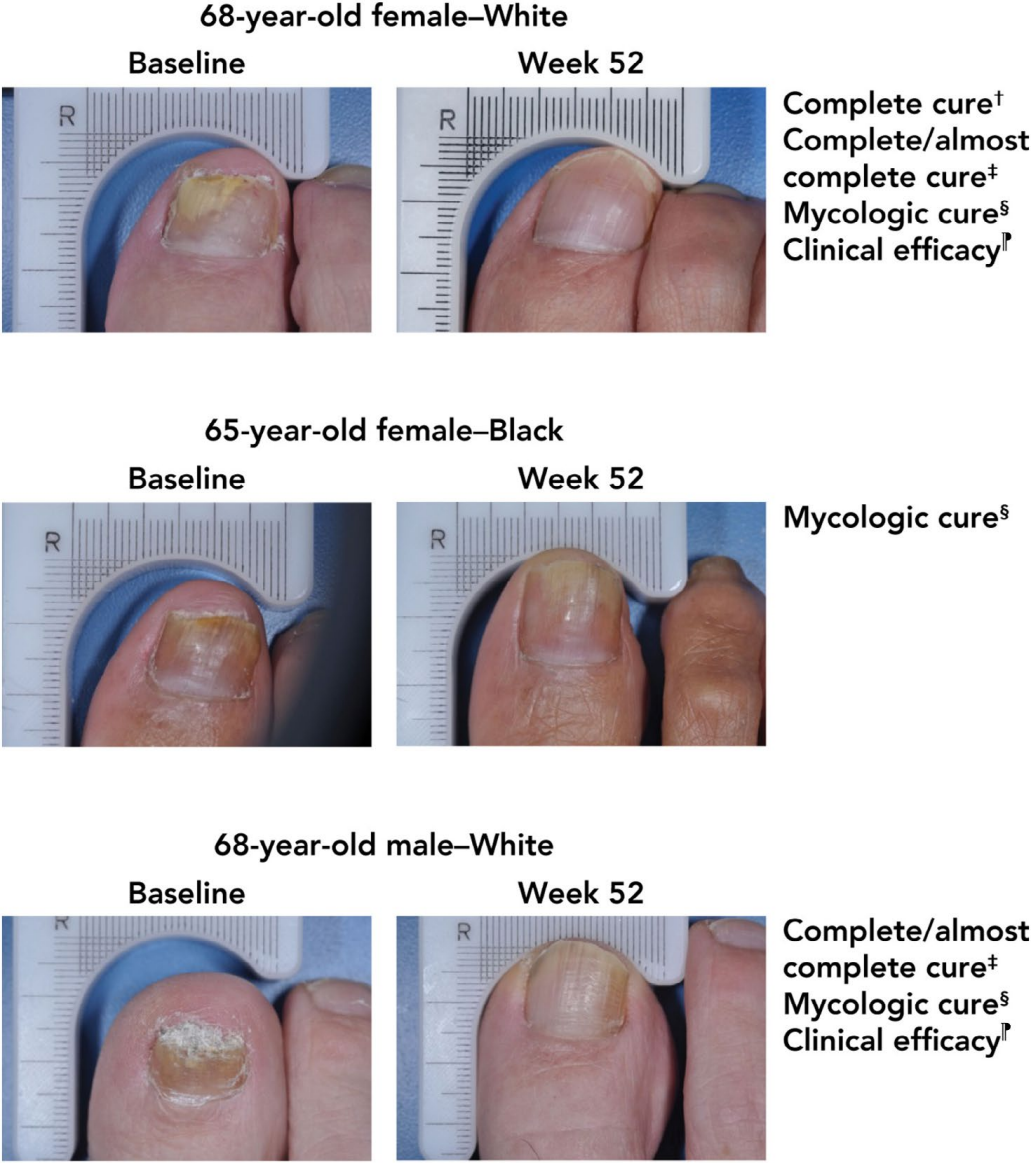
Representative photographs from three patients aged ≥ 65 years treated with efinaconazole 10% for 48 weeks. Individual results may vary. Photographic Images 2025^©^. Courtesy of Ortho Dermatologics Study Investigators. ^†^No clinical involvement of the target toenail and mycologic cure (negative KOH examination and negative fungal culture) of the target toenail. ^‡^≤ 5% clinical involvement and mycologic cure (negative KOH examination and negative fungal culture) of the target toenail. ^§^Negative KOH examination and negative fungal culture of the target toenail. ^⁋^< 10% involvement of the target toenail. KOH, potassium hydroxide.

At week 52, a significantly greater proportion of older adults treated with efinaconazole achieved complete cure versus vehicle (13.6% [*n*/*N* = 22/162] vs. 3.6% [2/56]; *p* < 0.05, Figure 1A). A significantly greater percentage of participants achieved complete or almost complete cure with efinaconazole compared with vehicle at week 52 (19.1% [31/162] vs. 5.4% [3/56]; *p* = 0.01), with numerically higher rates observed at weeks 36 and 48 (week 36: 9.3% [15/162] vs. 0% [0/56]; week 48: 18.5% [30/162] vs. 3.6% [2/56]; Figure 1B). Least squares mean unaffected new nail growth was significantly greater with efinaconazole versus vehicle at week 52 (3.9 mm vs. 0 mm; *p* < 0.001), and numerically greater with efinaconazole at all other time points (Figure 2). More than half of older adults (59.3% [96/162]) achieved mycologic cure with efinaconazole at week 52, a significantly greater percentage than with vehicle (12.5% [7/56]; *p* < 0.001; Figure 3). Almost half (49.4% [80/162]) of efinaconazole‐treated participants achieved mycologic cure as early as week 24. In addition, a quarter of participants treated with efinaconazole achieved clinical efficacy at week 52 (25.3% [41/162]) vs. 14.3% (8/56) of vehicle‐treated participants (*p* < 0.05; Figure 4). Representative photographs of affected toenails from older adults treated with efinaconazole are shown in Figure 5.

### 3.3 Quality of Life

The mean change from baseline in all OnyCOE‐t scale scores at weeks 24 and 52 was generally numerically greater (indicating improvement) among participants treated with efinaconazole than those treated with vehicle (Table 2).

**TABLE 2:** Mean (SD) change from baseline to weeks 24 and 52 in OnyCOE‐t^tm^ quality of life questionnaire in patients aged ≥ 65 years (ITT population, pooled).

Scale	Week 24		Week 52	
	Efinaconazole 10%	Vehicle	Efinaconazole 10%	Vehicle
Symptom frequency	28.3 (23.3)	23.2 (23.3)	25.0 (24.7)	19.5 (26.3)
Symptom bothersomeness	18.2 (21.2)	14.5 (23.5)	15.3 (19.1)	10.0 (28.5)
Physical activity problems	13.2 (20.8)	13.1 (28.7)	7.8 (23.5)	17.2 (27.4)
Appearance problems	17.0 (18.6)	17.6 (30.1)	14.3 (22.6)	17.6 (26.4)
Overall problem	17.0 (30.0)	15.3 (33.0)	16.1 (32.1)	19.6 (34.0)
Stigma	6.4 (16.5)	9.1 (21.0)	6.2 (21.2)	2.9 (21.3)
Treatment satisfaction	70.2 (30.1)	57.5 (33.0)	73.9 (31.9)	48.3 (37.4)

*Note:* Higher mean change scores indicate improvement.

### 3.4 Safety

The proportion of older adults who experienced treatment‐emergent AEs (TEAEs) through week 52 was slightly smaller with efinaconazole (67.9% [110/162]) than with vehicle (75.0% [42/56]). The majority of TEAEs were unrelated to the study drug (efinaconazole, 94.0%; vehicle, 98.6%; Table 3), most were mild to moderate in severity, and the discontinuation rate due to AEs was low (< 5%). The most common treatment‐related TEAE (reported in ≥ 3% of participants in any treatment group) with efinaconazole was application site dermatitis (4.3%).

**TABLE 3:** Treatment‐emergent adverse events through week 52 in patients aged ≥ 65 years (safety population, pooled).

Participants, *n* (%)	Efinaconazole 10% (*n* = 162)	Vehicle (*n* = 56)
Reporting any TEAE	110 (67.9)	42 (75.0)
Reporting any SAEs^a^	10 (4.0)	5 (3.5)
Discontinued drug or study due to AE	7 (4.3)	1 (1.8)
TEAE severity		
Mild	104 (41.9)	61 (43.0)
Moderate	130 (52.4)	75 (52.8)
Severe	14 (5.6)	6 (4.2)
Treatment‐related TEAEs	15 (6.0)	2 (1.4)
Most common treatment‐related TEAEs^b^		
Application site dermatitis	7 (4.3)	0

Abbreviations: AE, adverse event; SAE, serious adverse event; TEAE, treatment‐emergent adverse event.

^a^ None of the SAEs were considered related to the study drug.

^b^ Reported in ≥ 3% of participants in any treatment group.

## 4 Discussion

Topical efinaconazole 10% solution showed good efficacy in older adults aged 65–71 years with mild to moderate onychomycosis, with a significantly greater percentage of participants achieving complete, complete or almost complete, or mycologic cure versus vehicle at week 52. Additionally, the rates of treatment‐related TEAEs and discontinuations due to AEs were low with efinaconazole, demonstrating a good safety profile in this population.

Oral antifungal treatments are often the standard of care for toenail onychomycosis and have demonstrated efficacy in older adults. In the open‐label randomised IRON‐CLAD study of oral terbinafine (250 mg/d with or without debridement for 12 weeks), mycologic cure and complete cure were achieved in 64% and 28% of patients aged ≥ 65 years (*n* = 75), respectively, by week 48 [21]. In two studies of patients aged ≥ 60 years treated with terbinafine (*n* = 50 and 15; 250 mg/d for 12 weeks), mycologic cure rates at week 78 were 64.0% and 93.3% [22, 23]. One of these studies also included patients randomised to oral itraconazole (200 mg pulse; *n* = 51), with a mycologic cure rate of 62.7% at week 78; there were no significant differences in cure rates between terbinafine and itraconazole [23]. In a subgroup analysis of Japanese patients aged ≥ 65 years in a double‐blind, placebo‐controlled study of oral fosravuconazole (100 mg/d for 12 weeks; *n* = 39), a complete cure rate (0% clinical involvement and mycologic cure) of 64% was observed at week 48 [24]. It is important to note that while terbinafine, itraconazole, and fosravuconazole were generally efficacious in these older adult populations, some studies used less‐stringent definitions of mycologic cure (i.e., negative culture only [22] or negative KOH only [24] vs. negative culture plus negative KOH [21, 23]); as such, results should be interpreted with caution. Further, while these studies demonstrate the benefits of these treatments, the real‐world use of oral antifungals in older adults may be hindered by polypharmacy [25], contraindications and warnings/precautions (including black box warnings) [15, 16], poor treatment response resulting from impaired peripheral circulation to the nails [26], and lack of patient compliance [4], as well as rising rates of global antifungal resistance to terbinafine [12, 13]. To our knowledge, there are no published clinical trials of fluconazole or griseofulvin in the treatment of toenail onychomycosis in older adults.

Three topical agents have been approved in the US for the treatment of toenail onychomycosis: ciclopirox 8% lacquer, tavaborole 5% solution, and efinaconazole 10% solution [14]. In pivotal, phase 3, 52‐week clinical studies in patients with mild to moderate onychomycosis, higher rates of mycologic and complete cure were observed with efinaconazole (53.4% and 55.2%; 15.2% and 17.8%, respectively [19]) vs tavaborole (31.1% and 35.9%; 6.5% and 9.1% [27]) or ciclopirox (28.6% and 35.7%; 5.5% and 8.5% [28]). However, caution should be exercised when making comparisons across treatments, as these were not head‐to‐head studies. Efinaconazole has also demonstrated efficacy versus vehicle in pooled phase 3 post hoc analyses of patient sex, race/ethnicity, age, and baseline disease severity and in other special populations, including paediatric patients and patients with diabetes [29, 30, 31, 32, 33, 34, 35]. Unfortunately, there is a dearth of data on the efficacy of topical antifungals in older adults with onychomycosis. To our knowledge, only results for efinaconazole have been published [36, 37]; US prescribing information for tavaborole states that no overall differences in safety or effectiveness were observed between older and younger clinical study participants (though greater sensitivity in some older individuals cannot be ruled out) [38].

The analyses presented here demonstrated that almost 60% of older adults treated with efinaconazole, ranging in age from 65 to 71 years, achieved mycologic cure, and 13.6% achieved complete cure by week 52, which was similar to the overall populations in the two phase 3 studies (53.4% and 55.2%; 15.2% and 17.8%, respectively) [19]. This was also similar to mycologic cure rates seen in three of the four above‐mentioned studies of oral treatments in older patients (62%–64%), though definitions of mycologic cure differed across studies [21, 23, 24]. It is possible that higher cure rates could be achieved with continued treatment, as toenails can be slow to grow, especially in some older adults [2, 7]. In a 72‐week open‐label study of once‐daily efinaconazole in Japanese patients with severe onychomycosis, a subgroup of patients aged ≥ 65 years achieved a similar mycologic cure rate (60.2%) to that of the present analyses, but the complete cure rate (33.1%) was twice as high [36]. Similarly, a second 72‐week open‐label study of efinaconazole in Canada found mycologic cure rates of 57.9% in patients aged ≥ 70 years at 72 weeks of treatment [37]. Although older adults with onychomycosis are considered more difficult to treat, these results, together with those of the present analyses, demonstrate that topical efinaconazole is efficacious in older adults.

Moreover, despite age‐related increases in nail thickness and possible longer disease duration, the rates of efficacy with efinaconazole in older patients were similar to those in the overall phase 3 populations, which included participants as young as 18 years (overall mean age: 50.6 years) [19]. This most likely reflects the relatively low binding affinity of efinaconazole for keratin [39]. In vitro, efinaconazole has greater free‐drug concentration and keratin release after incubation in a keratin suspension than topical ciclopirox 8% or amorolfine 5% (not approved for use in the US) [39]. Efinaconazole 10% also has shown superior transungual penetration compared with tavaborole 5% and ciclopirox 8% [40].

In these pooled post hoc analyses, efinaconazole was also well tolerated in older adults, similar to the overall phase 3 populations. TEAEs occurred in 67.9% of older adults treated with efinaconazole versus 64.5% and 66.0% in overall populations, with low rates of discontinuations due to AEs (4.3% of older adults versus 3.2% and 1.9% of all patients) [19].

One possible limitation of the present studies is that treatment may need to be longer than 48 weeks to achieve higher rates of complete or almost complete cure in older adults due to slower nail growth. While longer treatment duration could potentially increase the risk of adverse events, a long‐term study of efinaconazole in Japan showed that rates of related TEAEs (6.4%) and discontinuations due to AEs (5.0%) at week 72 were similar to those seen at week 52 in the overall population of the phase 3 studies of efinaconazole (related TEAEs, 8.2% and 7.3%; discontinuations due to AEs, 3.2% and 1.9%) [19, 36]. Another 72‐week study of efinaconazole treatment in Canada showed no additional safety signals in patients ≥ 70 years of age [37]. An additional limitation of the p was the relatively short (4‐week) follow‐up period following treatment cessation. A follow‐up period longer than 4 weeks may also show greater efficacy rates. For example, further reductions in disease severity were observed in a small, open‐label study of 48 weeks of efinaconazole treatment with follow‐ups at 12 and 24 weeks post‐treatment [41]. Finally, it should be noted that the post hoc analyses presented here were not statistically powered for subgroup analyses.

## 5 Conclusion

Once‐daily topical efinaconazole 10% solution is a safe and effective treatment for toenail onychomycosis in patients aged ≥ 65 years with mild to moderate toenail distal lateral subungual onychomycosis. Efinaconazole showed similar efficacy and safety to the overall phase 3 populations [19], despite potential age‐related changes in nail growth or thickness in the older adult population. These results, taken together with those of other post hoc analyses, demonstrate the benefits of efinaconazole in various patient populations with onychomycosis, including male, female, and Hispanic patients, as well as those with longer disease duration or concurrent diabetes [42].

## Author Contributions

**Shari R. Lipner:** writing – review and editing, writing – original draft. **Aditya K. Gupta:** writing – original draft, writing – review and editing. **Warren S. Joseph:** writing – original draft, writing – review and editing. **Boni Elewski:** writing – original draft, writing – review and editing. **Eric Guenin:** writing – original draft, writing – review and editing. **Tracey C. Vlahovic:** writing – review and editing, writing – original draft.

## Disclosure

Shari R. Lipner has served as a consultant for Moberg Pharmaceuticals and BelleTorus Corporation. Aditya K. Gupta has served as a consultant, speaker, and investigator for Ortho Dermatologics. Warren S. Joseph has served as a consultant and speaker for Ortho Dermatologics. Boni Elewski has provided clinical research support (research funding to University) for AbbVie, Anaptys‐Bio, Boehringer Ingelheim, Bristol Myers Squibb, Celgene, Incyte, LEO Pharma, Lilly, Merck, Menlo, Novartis, Pfizer, Regeneron, Sun Pharma, Ortho Dermatologics, and Vanda and was a consultant (received honorarium) for Boehringer Ingelheim, Bristol Myers Squibb, Celgene, LEO Pharma, Lilly, Menlo, Novartis, Pfizer, Sun Pharma, Ortho Dermatologics, and Verrica. Eric Guenin is an employee of Ortho Dermatologics and may hold stock and/or stock options in its parent company. Tracey C. Vlahovic has served as an investigator and speaker for Ortho Dermatologics.

## Ethics Statement

The authors confirm that the ethical policies of the journal, as noted on the journal's author guidelines page, have been adhered to, and the appropriate ethical review committee approval has been received.

## Conflicts of Interest

The authors declare no conflicts of interest.

## Data Availability

The data that support the findings of this study are available from the corresponding author upon reasonable request.
